# Incorporation of patient and public involvement in statistical methodology research: development of an animation

**DOI:** 10.1186/s40900-023-00513-7

**Published:** 2023-11-08

**Authors:** Hannah M. Worboys, Jonathan Broomfield, Aiden Smith, Rachael Stannard, Freya Tyrer, Elpida Vounzoulaki, Barbara Czyznikowska, Gurpreet Grewal-Santini, Justin Greenwood, Laura J. Gray

**Affiliations:** 1 Department of Population Health Sciences, University of Leicester, Leicester, UK; 2 Leicester Real World Evidence Unit, Diabetes Research Centre, University of Leicester, Leicester, UK; 3 Centre for Ethnic Health Research, University of Leicester, Leicester, UK; 4 Public Contributor, PPI-SMART (Public and Patient Involvement in Statistical Methods and Research Techniques), University of Leicester, Leicester, UK

**Keywords:** Patient and Public Involvement and Engagement (PPIE), Statistical methodology research, Communication, Visualisation, Animation, Involvement, Public health

## Abstract

**Background:**

Patient and Public Involvement and Engagement (PPIE) is important to all aspects of health research. However, there are few examples of successful PPIE in statistical methodology research. One of the reasons for this relates to challenges in the identification of individuals interested in statistical methodology research projects, and ambiguities over the importance of PPIE to these projects.

**Methods:**

This project was conducted between August 2022 and August 2023. The aim is to report the process of the development of an accessible animation to describe what statistical methodology is and the importance of PPIE in statistical methodology research projects. For this, we combined storyboarding and scriptwriting with feedback from PPIE members and researchers.

**Results:**

After three stages that incorporated feedback from the relevant stakeholders, we produced a final animation about PPIE in statistical methodology. The resulting animation used minimal text, simple animation techniques and was of short duration (< 3 min) to optimise the communication of the key messages clearly and effectively.

**Conclusions:**

The resulting animation provides a starting point for members of the public to learn about PPIE in statistical methodology research and for methodologists who wish to conduct PPIE. We recommend further work to explore ways in which members of the public can be more meaningfully involved in methodology research.

**Supplementary Information:**

The online version contains supplementary material available at 10.1186/s40900-023-00513-7.

## Abstract (plain language summary)

Patient and public involvement and engagement (PPIE) is when members of the public are directly involved in carrying out research projects. This is important because we as researchers want to make sure we are focusing on what matters most to patients, so that the research has as large an impact as possible. PPIE has typically been used in more applied research projects, such as clinical trials, but is equally as important in statistical methodology research, where we focus on making sure the statistical tools that we use in the applied projects are as good as possible. The aim of this project was to create a short animation that helps to explain the importance of PPIE in statistical methodology research projects. Researchers sometimes incorrectly assume that PPIE is less important in these projects as this type of research has a less obvious benefit to patients. The animation helps to further explain these concepts. It describes what statistical methodology research is and why involving members of the public is still important. This paper explains the process of developing the animation, including receiving feedback from members of the public to make sure the animation is accessible to as many people as possible. The result is a short, 3-min animation that is free to view on the NIHR website. This can be used by other researchers to help them when recruiting members of the public to their research projects.

**Supplementary Information:**

The online version contains supplementary material available at 10.1186/s40900-023-00513-7.

## Background

Patient and public involvement and engagement (PPIE) in research has become increasingly immersed within health research and plays a key role in the improvement of its quality, relevance, and appropriateness. It primarily concerns research being conducted ‘with’ or ‘by’ patients and members of the public rather than ‘to’, ‘about’ or ‘for’ them [1]. PPIE covers a diverse range of approaches, from one-off information gathering such as a single project proposal meeting, to sustained partnerships such as regular meetings throughout the duration of a project. Patients and members of the public can shape research studies through various stages of a project, from initial planning through to dissemination of results. Their involvement can be high (‘user-led’), where PPIE members are actively involved in decision-making processes throughout the project, or low (‘consultation’) where their involvement is restricted to providing feedback on a project plan [2, 3]. In recent years, PPIE has become a key component of funding applications, research proposals and ethics applications [4].

For applied research studies, we have seen a rise in PPIE activity in recent years, including examples of where PPIE is effective and meaningful [5]. In part, this is a result of funding body initiatives, such as the National Institute for Health and Care Research (NIHR), who promote PPIE across all study designs—but also because it is increasingly recognised that patients can provide unique insights that add value to the overall study [6, 7]. Through engaging with patients and the public, researchers can ensure that they are answering relevant questions in the areas of greatest importance to those it impacts the most. This could be those with a specific health condition, or those in underserved groups who experience health inequalities due to a lack of representation in research. Similarly, in scenarios where the study population is not adequately represented by members of the research team, it is important to obtain relevant perspectives from members of those communities. Patients and the public can also benefit substantially from being involved in studies, leading to empowerment and a better understanding and management of their own conditions.

Many health research funders stipulate that PPIE should be incorporated at both the design and implementation stages of research. This applies not only to applied research, but also to methodology research—which includes statistical methodology research. Typically, applied research concerns the study of specific health conditions, interventions, and exposures, whereas statistical methodology research concerns the development, evaluation or comparison of methods for designing studies, analysing data, and presenting results. The aim is to identify the most appropriate statistical tools to ensure a research question is answered in an appropriate and meaningful way, subsequently improving patient care and outcomes. PPIE is much less common in statistical methodology research partly because it may be one step removed from patients and the public and has less discernible direct patient impact. Incorporating PPIE can be more challenging in this type of research since research goals may be deemed more abstract than those seen in applied studies [8].

Several barriers exist to conducting PPIE in statistical methodology research. Researchers may feel unprepared to facilitate meaningful PPIE if they themselves do not understand how patients and the public can engage with and aid in shaping their research. A lack of communication and understanding between researchers and patients and the public regarding the concept of statistical methodology can hinder not only the recruitment process but also the scope for patients and the public to shape the research. This has the potential to encourage tokenistic PPIE inclusion to satisfy funders, rather than meaningful PPIE to improve research quality. The development and dissemination of resources specially designed to improve understanding of statistical methodology to patients and the public could help to alleviate this barrier.

## Aims

The overarching aim of this work was to develop an animation for members of the public to support and improve their knowledge of statistical methodology research and highlight the importance of their input. A secondary aim was to develop a resource for statistical methodologists to disseminate to potential public contributors.

## Methods

### Setting and PPIE groups

This project ran from July 2022 to August 2023 and was conducted in the Biostatistics Research Group within the Department of Population Health Sciences, University of Leicester, UK. We involved two PPIE groups in the process of developing this animation. The first (**‘PPIE Group 1’**) comprised six members (n = 3 male; n = 3 female; all from ethnic minority backgrounds) of an established PPIE group at the Centre for Ethnic Health Research, Diabetes Research Centre. The second group (**‘PPIE Group 2’**) was recruited through the ‘People in Research’ website run by the NIHR Centre for Engagement and Dissemination, UK. This latter group comprised seven individuals: one male and six females (4 from minority ethnic backgrounds). The GRIPP2 (Guidance for Reporting Involvement of Patients and the Public) checklist [9] was followed and completed (see Additional file 2).

### Development of the animation

**Fig. 1:**
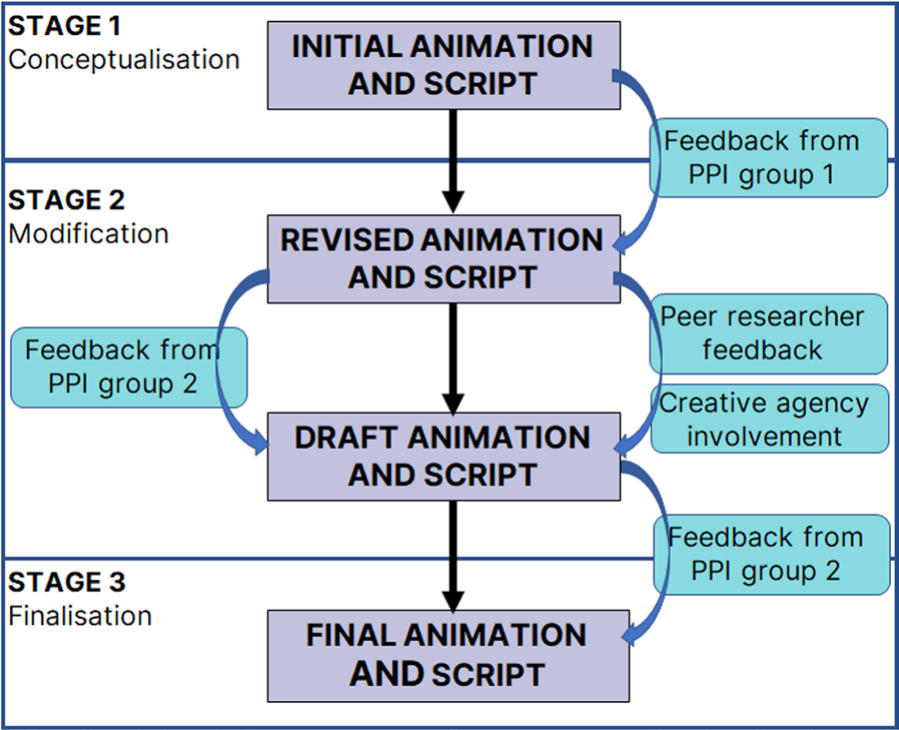
Flow diagram of process of developing the animation for PPI in statistical methodology research

The steps taken in the animation development are summarised in Fig. 1. Having agreed on the rationale and need for the work, a group of 12 statistical methodologists discussed developing an animation that incorporated key messages and terminology in relation to statistical methodology research. Through a process of brainstorming and group discussions, we defined statistical methodology research in simple terms, and described the importance of involving PPIE.

### Stage 1: Conceptualisation

Members of the team drafted a script using an example to help to explain statisitical methodology research: this involved two researchers discussing the recent results of a clinical trial and one highlighting an error in the method used by the another. We used a graph, with red and blue drugs for further clarification. The narrative then moved onto a voiceover by a third person to explain how the incorrect use of methods can lead to suboptimal outcomes for patients, and how statistical methodology research is needed to identify best methods (see Additional file 1 for the initial script and subsequent iterations). Once the script was finalised, we had a meeting with **PPIE Group 1** to receive feedback about the content of the script.

All PPIE members reported that they struggled to relate to the script and were unsure where they fitted into the animation. One member highlighted the need to be clear about the target audience and why PPIE input was necessary. Some members felt that we needed to clarify that the animation was describing an area of research, rather than a particular project. The group all stated that we should be clearer about why statistical methodology research is important, also giving more context to the script. Some members found the ‘red versus blue’ group analogy hard to visualise or felt that it was confusing. They also struggled to see why the script represented two researchers and not PPIE contributors. They proposed the inclusion of multiple patient groups to increase representation, particularly of minority ethnic communities. Accessibility and language were also discussed in the context of research jargon used in the script, with most feeling that accessibility was limited, and few would be able to understand the proposed animation. Following feedback from **PPIE Group 1**, we realised that the example of a methodological problem in a trial-based setting was too complex. Moreover, the use of researchers to describe statistical methodology was not appropriate. The feedback received from this group clearly highlights the importance of PPIE, as our thoughts on what was being described was widely different from our target audience’s.

### Stage 2: Modification

In response to the comments from **PPIE Group 1**, we moved away from a two-person dialogue format to a narrated piece. We also proposed a more abstract visualisation on the screen that matched the narration, rather than a story-telling scene format.

We revised the narrative to describe statistical methodology, restructuring the example to a metaphorical representation of the type of problems we encounter to avoid the need to explain any statistical concepts. The metaphor we chose was hanging a picture on a wall with the correct tool (i.e. a hammer, rather than a shoe or glass hammer for example) to avoid ‘unintended outcomes’ and reach’the desired outcome quickly and easily’.

We circulated our second draft to colleagues (non-statistical specialists) in the department of Population Health Sciences and presented our findings at our annual Departmental Conference. Overall, they gave positive feedback to this updated analogy but made minor comments on how to make the narrative clearer. By removing a statistical example, readers were able to focus more clearly on the purpose of the animation—to explain the concept of statistical methodology research to the public and identify where they could assist with this research.

After this stage, we considered more innovative and creative ways of communicating with the public, particularly with regard to representing them more fully as making a valuable contribution to the statistical methodology process. Four members of **PPIE Group 2** (all on-line: n = 2 individually; n = 2 in a group setting) also gave feedback about the animation ideas.

Given the complexity of the animation and importance of good communication, we approached a creative agency who have a vast experience of working with academia to make our project more visually engaging, communicative and impactful. Four researchers and two community engagement officers worked with the agency to brainstorm ideas for the animation. We showed the agency our revised script and feedback that we had already received. As part of the brainstorming session for the animation, we focused on four main components; who was the target audience for the animation; why we were creating the animation; what was the context, problem and solution; and a call to action on how the animation was solving the problem identified.

The audience for the animation is prospective PPIE members with little or no experience in statistical methodology research. In applied research, the PPIE group typically comprise people living with a specific condition. This is not important for statistical methodology research so the animation needed to be more general and targeted to anyone interested. We discussed a secondary target audience for our animation—statistical methodologists who could distribute the animation when identifying their own PPIE contributors. The multiple reasons for involving PPIE in our project is already described in section "Background". We also discussed our own difficulties, as methodologists, in conducting PPIE for methodological research. We explained to the design team the difference that PPIE could make to projects and gave some examples of where it had been useful. This allowed the designers to understand the importance of the animation, and its role in improving the quality of research.

The design team were provided with our final script and modified the content—in particular the metaphor that was used. The original ‘hammer idea’ was changed to ‘digging a hole’ as an easier visual metaphor that was understood across diverse communities. The final animation script is given in Box 1.

**Box 1:** Final animation script to introduce PPIE in statistical methodology

“Did you know that the tools we use to look at numbers can change the world?
How we collect, look at and present numbers—or data—shape how we answer research questions. Questions like how we treat cancer right through to climate change!
People who use numbers like this are called statisticians. They use maths techniques, theories and models to analyse data to see what it tells us. This collection of tools is called statistical methodology
Statistical methodologists explore which tools work best when analysing data
It’s a bit like finding the right tool to dig a hole. The best tool will depend on many things, like how big a hole is needed or how much time we have. You could use a spoon, but it would take too long and not do a good job. You could use a mechanical digger, but this might make the hole TOO big and damage other things outside the hole. A spade is the best bet!
Statistical methodologists make sure that the spade—or the mathematical tools—are the best, quickest and most appropriate way to dig the hole—or collect, analyse and apply data to a research question. The better the tools, the more likely the data will make a difference to real life, like improving patient care
This is where members of the public like YOU come in
It might not seem obvious how you can help—it could sound like scary maths! But don’t worry, you don’t have to do or know any maths at all!
You can help statistical methodologists build and select tools that are appropriate to the research topic. This is because your lived experience and knowledge of the research topics means that you can tell statisticians what's most important to look at, where they need to collect data, and what they are missing. Or, what kind of holes they need to dig, where and how deep!
Your feedback on the tools for data analysis for one study, could change the ENTIRE way that data is analysed across that area of research
Help us build statistical tools that could change the world
To find out more about public involvement in statistical methodology research, visit the link on-screen.”

After agreeing on the final script, we made decisions on the colour scheme and other design aspects for the animation, choosing to adopt the colour scheme outlined by the NIHR [10]. The designers then created a story board of 14 frames of still graphics for review along with a description of how the graphics would be animated in the final version. The statistical research team reviewed and requested changes to the storyboard before putting the animation together. The designers then animated the storyboard and combined it with a voiceover of the script to provide an animation, which we reviewed again and collated comments before seeking feedback from the PPIE group.

### Stage 3: Finalisation

**PPIE Group 2** gave feedback on the draft animation (online: 6 individuals: n = 1 individually, n = 1 by email and n = 4 in a group setting). The group had limited PPIE experience in statistical research. Those that have previously worked with statisticians understood the difficulties in communicating with the public effectively. Overall, the feedback was extremely positive: members liked the animation and felt the descriptions of terms were clear and concise. The length was deemed to be appropriate (2 min and 34 s) but should not be made any longer to avoid ‘losing the audience’. A short accompanying introduction was recommended to explain the purpose of the animation in simple terms (we confirmed that we intended to do this via the animation website). Some PPIE members felt that some floating text may benefit certain sections to highlight key terms such as’statistics’ and 'public’. Finally, the group highlighted that the benefits to PPIE contributors themselves should be stressed as well as the benefits to the researchers.

## Conclusions

This project highlights that it is possible to describe statistical methodology to members of the public in an accessible way, with careful thought and planning. The final animation has been published on the NIHR Learning for Involvement website, please see section "Access to animation" for the website information. Researchers are welcome to use these resources to aid with their PPIE work. The animation has had 460 views within the first two months of being published.

With the growing importance of PPIE in all research studies, not just applied research, our animation provides an ideal starting point for methodologists who wish to conduct PPIE but do not know where to start. It also benefits members of the public as they can learn about statistical methodology and understand its role in shaping applied health research.

This project had some limitations that need to be acknowledged. First, we had a limited budget for this work so could not spend as much time with our PPIE contributors as we would have liked. Having additional time to further refine the animation would likely have benefited the project by making the animation even more relevant to the target audience. Secondly, this project does not address some of the challenges faced by statistical methodologists nor provide any training in how best to communicate with members of the public. The ability to adapt our communication skills to different audiences is essential if we are to convey information about our research and their findings effectively. Training and development in communication skills is likely to be a priority moving forward. Other than the access to, and subsequent feedback from PPIEE contributors about, our animation, we recognise that are not able to evaluate the impact of PPIEE in this study which is a recognised challenge in PPIEE work [3]. We did not formalise the development process because we wanted to create a fluid dialogue between PPIEE contributors and researchers, but we recognise that this prevented us from doing more robust quantitative evaluation of when and how improvements and modifications were implemented.

Finally, this is the first stage of a longer-term strategy to improve and facilitate public involvement in statistical methodology research. Future work will include the development of a second animation to outline successful case studies of PPIE involvement in methodological studies. We also intend to develop guidance for methodologists who are aiming to conduct meaningful PPIE, particularly for the first time. The lack of such guidance has been identified as a barrier to conducting PPIE even when the benefits are known [8]. We look forward to continued work in this area to enable statistical methodologists and members of the public to work collaboratively with one another, which will ultimately lead to greater satisfaction and patient benefit.

## Access to animation

The animation is on the NIHR Learning for Involvement website: https://www.learningforinvolvement.org.uk/content/resource/what-is-statistical-methodology-research-and-why-is-ppie-input-important/.

The animation can be viewed directly here: https://www.youtube.com/watch?v=4rzEHbA4p48.

### Supplementary Information

**Additional file 1**. Iterations of the animation scripts.

**Additional file 2**. **Table 1:** GRIPP2 Long Form.

## Data Availability

Not applicable.

## Abbreviations

PPIE: Patient and Public Involvement
NIHR: National institute for Health and Care Research
GRIPP: Guidance for Reporting Involvement of Patients and the Public
