# Supplementary material for: Incorporation of patient and public involvement in statistical methodology research: development of an animation
Source: Res Involv Engagem. 2023 Nov 8;9:102. doi: 10.1186/s40900-023-00513-7 (PMC10631193; doi:10.1186/s40900-023-00513-7)
Supplement: Supplementary file 1 — Additional file 1. Iterations of the animation scripts. [file 40900_2023_513_MOESM1_ESM.docx]

**Supplementary material 1: Iterations of the animation scripts**

The iterations of the animation scripts are presented in turn.

Iteration 1

The first script iteration describes two researchers (R1 and R2) discussing an example of a methodological issue than may be encountered in statistical methodological research, before the scene changes to a third researcher (R3) explaining the research more generally and the motivation behind such research.

*Scene 1: Two researchers sat at their computers. One turns to the other.*

*R1: Just got the results from my new clinical trial.*

*R2: Is that the one where you were comparing two drugs to see which one gave participants a better quality of life?*

*R1: Yeah - it seems like the blue drug is much better, look how much higher their quality of life is compared to the red drug group!*


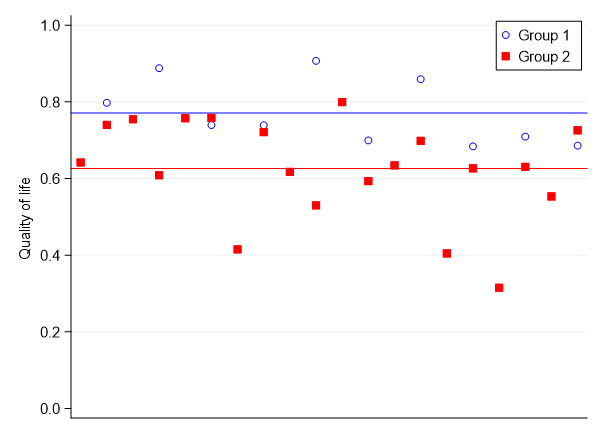


*R2: Wow, that’s pretty clear-cut! But hold on, why are there more red squares than blue circles? I thought there were the same number of participants in each group.*

*R1: Oh, there were when we started the trial, but lots of people dropped out from the blue group because of side-effects. I only included participants in the final analysis if they didn’t drop out.*

*R2: Hmmmmm…I know you can do that in some scenarios, but I think in this case there might be a problem. Isn’t it possible that the participants with the blue drug that dropped out had a worse quality of life because they were experiencing side effects?*

*R1: Uhhhh, yeah I guess so.*

*R2: Well then, if you had their data to include in your analysis, then the quality of life in the blue group would go down wouldn’t it?*

*R1: Ahhhh, I see. So the blue drug might not be any better than the red drug - it might just be because I don’t have the data for the participants in the blue group that experienced side effects?*

*R2: Exactly!*

*R1: So what can I do? What method should I use?*

*Freeze scene and cut to scene 2.*

*Scene 2: A third researcher talks to the camera.*

*R3: Problems like this arise all the time in medical research.  Statistical methods are the tools we use to analyse data to answer a research question.  We need to make sure we use the best method to get the right answer from our data.  Sometimes more than one method exists and we need to choose the best one, other times we can adapt an existing method or develop a completely new one. This is what we mean by statistical methods research. By finding the best method to use, we can be sure that the research question is being answered correctly, which will lead to improvements in public health.*

*Scene 3:*


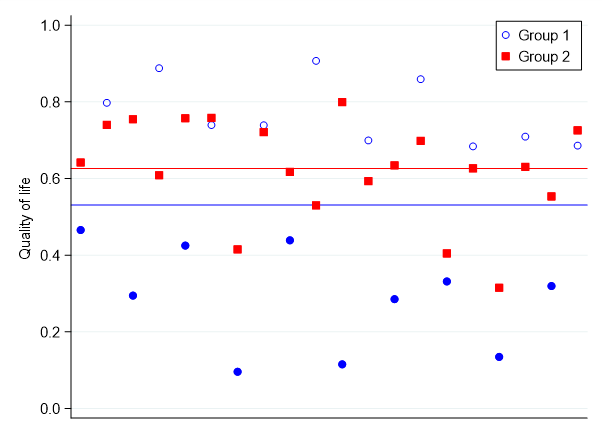


*R1: Well, now that I’ve accounted for the participants in the blue group that dropped out, it seems like the red group have better quality of life.*

*R2: Glad I could help. Now, if you could just help me with my data, I’ve never analysed questionnaires before and there seems to be lots of different methods…*

Iteration 2

The second script iteration was a narrated piece, describing statistical methodological research through a more abstract metaphor of hanging a picture on a wall.

***Introduction to Statistical Methodology Research***

*What is statistical methodology research?*

*Statistics is a really important part of research, statisticians help design and analyse data from research studies.*

*But how do statisticians know the best way to design and analyse these studies?*

*This is where statistical methodology research comes in. This type of research makes sure we use the right statistical tools to ensure our study has the best chance of answering our research question and therefore improving patient care and outcomes.*

*Imagine trying to hang a picture on the wall with a glass hammer. In statistics if we pick the wrong tool for the job our analysis won’t provide the correct answer to the research question, which wastes time and resources.*

*We could try hanging the picture with a shoe – this would do the job but we might end up with unintended outcomes, such as a dirty wall or a broken shoe. In statistics, sometimes the tools we have are not as good as they could be and we can do statistical methodology research to try and improve them.*

*Using a proper hammer to hang the picture gets the desired outcome quickly and easily. If we can develop better statistical tools, this can speed up the research process, so we get our research question answered sooner than we would have done if we used a tool which wasn’t as good. This is good for patients as it means new treatments get into practice quickly.*

*Patient and public involvement is important in both designing and carrying out research.*

Iteration 3

The third and final script iteration was still a narrated piece, but replaced the picture on a wall metaphor with a person digging a hole. Patient and Public Involvement (PPI) was also introduced at the end of the script.

*“Did you know that the tools we use to look at numbers can change the world?*

*How we collect, look at and present numbers - or data - shape how we answer research questions.*

*Questions like how we treat cancer right through to climate change!*

*People who use numbers like this are called statisticians. They use maths techniques, theories and models to analyse data to see what it tells us. This collection of tools is called statistical methodology.*

*Statistical methodologists explore which tools work best when analysing data.*

*It’s a bit like finding the right tool to dig a hole. The best tool will depend on many things, like how big a hole is needed or how much time we have. You could use a spoon, but it would take too long and not do a good job. You could use a mechanical digger, but this might make the hole TOO big and damage other things outside the hole. A spade is the best bet!*

*Statistical methodologists make sure that the spade - or the mathematical tools - are the best, quickest and most appropriate way to dig the hole - or collect, analyse and apply data to a research question. The better the tools, the more likely the data will make a difference to real life, like improving patient care.*

*This is where members of the public like YOU come in.*

*It might not seem obvious how you can help - it could sound like scary maths! But don’t worry, you don’t have to do or know any maths at all!*

*You can help statistical methodologists build and select tools that are appropriate to the research topic. This is because your lived experience and knowledge of the research topics means that you can tell statisticians what's most important to look at, where they need to collect data, and what they are missing. Or, what kind of holes they need to dig, where and how deep!*

*Your feedback on the tools for data analysis for one study, could change the ENTIRE way that data is analysed across that area of research.*

*Help us build statistical tools that could change the world.*

*To find out more about public involvement in statistical methodology research, visit the link on-screen.”*
